# Cardiac noradrenergic deficiency revealed by ^18^F-dopamine positron emission tomography identifies preclinical central Lewy body diseases

**DOI:** 10.1172/JCI172460

**Published:** 2024-01-02

**Authors:** David S. Goldstein, Courtney Holmes, Patti Sullivan, Grisel Lopez, Janna Gelsomino, Sarah Moore, Risa Isonaka, Tianxia Wu, Yehonatan Sharabi

**Affiliations:** 1 Autonomic Medicine Section, Clinical Neurosciences Program, Division of Intramural Research (DIR), National Institute of Neurological Disorders and Stroke (NINDS),; 2 Molecular Neurogenetics Section, National Human Genome Research Institute, and; 3 Clinical Trials Unit, Office of the Clinical Director, DIR, NINDS, NIH, Bethesda, Maryland, USA.; 4 Chaim Sheba Medical Center, Tel-Aviv University, Tel-Hashomer, Israel.

**Keywords:** Neuroscience, Neurodegeneration, Neuroimaging, Parkinson disease

## Abstract

**BACKGROUND.** In Lewy body diseases (LBDs) Parkinson disease (PD), and dementia with Lewy bodies (DLB), by the time parkinsonism or cognitive dysfunction manifests clinically, substantial neurodegeneration has already occurred. Biomarkers are needed to identify central LBDs in a preclinical phase, when neurorescue strategies might forestall symptomatic disease. This phase may involve catecholamine deficiency in the autonomic nervous system. We analyzed data from the prospective, observational, long-term PDRisk study to assess the predictive value of low versus normal cardiac ^^18^^F-dopamine positron emission tomography (PET), an index of myocardial content of the sympathetic neurotransmitter norepinephrine, in at-risk individuals.

**METHODS.** Participants self-reported risk factor information (genetics, olfactory dysfunction, dream enactment behavior, and orthostatic intolerance or hypotension) at a protocol-specific website. Thirty-four with 3 or more confirmed risk factors underwent serial cardiac ^^18^^F-dopamine PET at 1.5-year intervals for up to 7.5 years or until PD was diagnosed.

**RESULTS.** Nine participants had low initial myocardial ^^18^^F-dopamine–derived radioactivity (<6,000 nCi-kg/cc-mCi) and 25 had normal radioactivity. At 7 years of follow-up, 8 of 9 with low initial radioactivity and 1 of 11 with normal radioactivity were diagnosed with a central LBD (LBD+) (*P* = 0.0009 by Fisher’s exact test). Conversely, all 9 LBD+ participants had low ^^18^^F-dopamine–derived radioactivity before or at the time of diagnosis of a central LBD, whereas among 25 participants without a central LBD only 1 (4%) had persistently low radioactivity (*P* < 0.0001 by Fisher’s exact test).

**CONCLUSION.** Cardiac ^^18^^F-dopamine PET highly efficiently distinguishes at-risk individuals who are diagnosed subsequently with a central LBD from those who are not.

**TRIAL REGISTRATION.** ClinicalTrials.gov NCT00775853.

**FUNDING.** Division of Intramural Research, NIH, NINDS.

## Abstract 2

A prospective, observational, long-term study reveals cardiac 18F-dopamine positron emission tomography as a strategy to identify preclinical central Lewy body diseases in at-risk individuals.

## Introduction

Aging-related neurodegenerative diseases are posing an increasing public health burden as populations are getting older. Prominent among these conditions are the central Lewy body diseases (LBDs), Parkinson disease (PD), and dementia with Lewy bodies (DLB).

By the time symptoms or signs of a central LBD develop, it is likely that substantial central neurodegeneration has already occurred. There is an urgent need for biomarkers that can detect the pathophysiological process in a preclinical phase, when strategies such as lifestyle modifications, dietary supplements, or prophylactic medications might delay the onset of, or even prevent, symptomatic disease.

PD and DLB entail cardiac sympathetic denervation (1–4). Indeed, in PD there is as much depletion of the sympathetic neurotransmitter norepinephrine in the left ventricular myocardium (5) as there is of the closely related catecholamine dopamine in the putamen (6), the main site of catecholamine deficiency in the brain. DLB involves at least as frequent cardiac noradrenergic deficiency as does PD (7, 8). Except in isolated cases (9, 10), however, whether cardiac noradrenergic deficiency precedes central LBDs has been unknown. Thus, although it is widely suspected that the pathogenetic process leading to PD can begin outside the brain with early involvement of the autonomic nervous system (11–14), to date this key issue has not been addressed directly in a prospective, long-term follow-up study.

The intramural NINDS PDRisk study (ClinicalTrials.gov NCT00775853) was designed to determine whether in people with multiple PD risk factors biomarkers of catecholamine deficiency in the heart or brain predict PD during long-term follow-up. Entry into the PDRisk study was based on 4 categories of risk — genetic, olfactory, dream enactment behavior (as in rapid eye movement behavior disorder [RBD]), and orthostatic intolerance or orthostatic hypotension (OH), all of which have been reported to precede the onset of both PD (15–18) and DLB (19–23).

A “first look” at the data (24) revealed that in at-risk individuals, low myocardial concentrations of the sympathetic neuroimaging agent ^^^18^^^F-dopamine quantified by positron emission tomography (PET), a validated biomarker of depleted myocardial norepinephrine stores (25), predicted PD at 3 years of follow-up. At this early point the study did not address the meaning of persistently negative biomarkers or conversion from negative to positive biomarkers during follow-up; the possible spatiotemporal sequence from an early cardiac sympathetic to a later nigrostriatal dopaminergic lesion; or mechanisms of cardiac noradrenergic deficiency, which as explained below are relevant to potential experimental neuroprotective trials.

The results of the initial report justified continuation and expansion of the study, with more participants followed for longer time periods (up to 7.5 years, with inpatient evaluations every approximately 1.5 years), in order to assess the relative risks associated with low versus normal ^^^18^^^F-dopamine–derived radioactivity. Accrual into the PDRisk study and follow-up testing have been completed. Because the same risk factors apply to DLB, the analysis was extended to DLB as an outcome measure.

In addition to ^^^18^^^F-dopamine PET, participants underwent brain ^^^18^^^F-DOPA PET to assess the integrity of putamen dopaminergic terminals (26, 27), with magnetic resonance imaging for anatomic registration of ^^^18^^^F-DOPA PET scans in the same individuals using the PMOD image analysis package (28). Other tests included ^^^13^^^N-ammonia PET to adjust ^^^18^^^F-dopamine–derived radioactivity for myocardial perfusion (29); lumbar puncture for assaying cerebrospinal fluid (CSF) levels of catechols such as 3,4-dihydroxyphenylacetic acid (DOPAC), the main neuronal metabolite of dopamine (30); and physiological and neurochemical autonomic function testing (31–33). The serial nature of the PET imaging enabled examination of whether cardiac noradrenergic abnormalities precede nigrostriatal dopaminergic abnormalities, as one would expect if central LBDs entailed early involvement of the sympathetic innervation of the heart (12, 13).

To examine mechanisms of cardiac noradrenergic deficiency, we assessed indices of denervation and dysfunction in residual nerves (34). Distinguishing between the 2 determinants of cardiac noradrenergic deficiency is relevant to potential experimental therapeutic trials, because, put bluntly, you cannot treat neurons that are dead, whereas neurons that are dysfunctional but alive (5) might be rescued (35).

The term “preclinical” is used here to denote insufficient initial evidence to diagnose a parkinsonian movement disorder, coupled with a subsequent diagnosis of a central LBD (PD or DLB). The MDS Research Criteria for prodromal PD, as updated in 2019 (36), include RBD, olfactory dysfunction, and clinical symptoms of dysautonomia.

We paid particular attention to the possibility of decreased efficiency of vesicular sequestration of cytoplasmic catecholamines via the type 2 vesicular monoamine transporter (VMAT2). A vesicular storage defect would not only deplete releasable neurotransmitter stores but also promote autotoxicity from augmented oxidation of cytoplasmic catecholamines (37–39). Diminished vesicular storage in existing nerve terminals could have any of several causes, such as decreased mitochondrial complex 1 activity (40), α-synuclein (αS) oligomers (37, 41), or decreased VMAT2 availability (42), all of which are potential therapeutic targets; however, no previous study has determined whether indices of attenuated vesicular sequestration in cardiac sympathetic nerves precede central LBDs in at-risk individuals.

In summary, in this prospective, observational, long-term follow-up study we asked whether among individuals with multiple PD risk factors cardiac sympathetic denervation or dysfunction revealed by ^^^18^^^F-dopamine PET separates the group subsequently diagnosed with a central LBD (LBD+) from the group not so diagnosed (LBD–). The study also addressed whether the results of serial ^^^18^^^F-dopamine and ^^^18^^^F-DOPA PET support a sequence of pathophysiologic progression from cardiac noradrenergic to putamen dopaminergic deficiency.

## Results

### Participant accrual.

**Figure 1:**
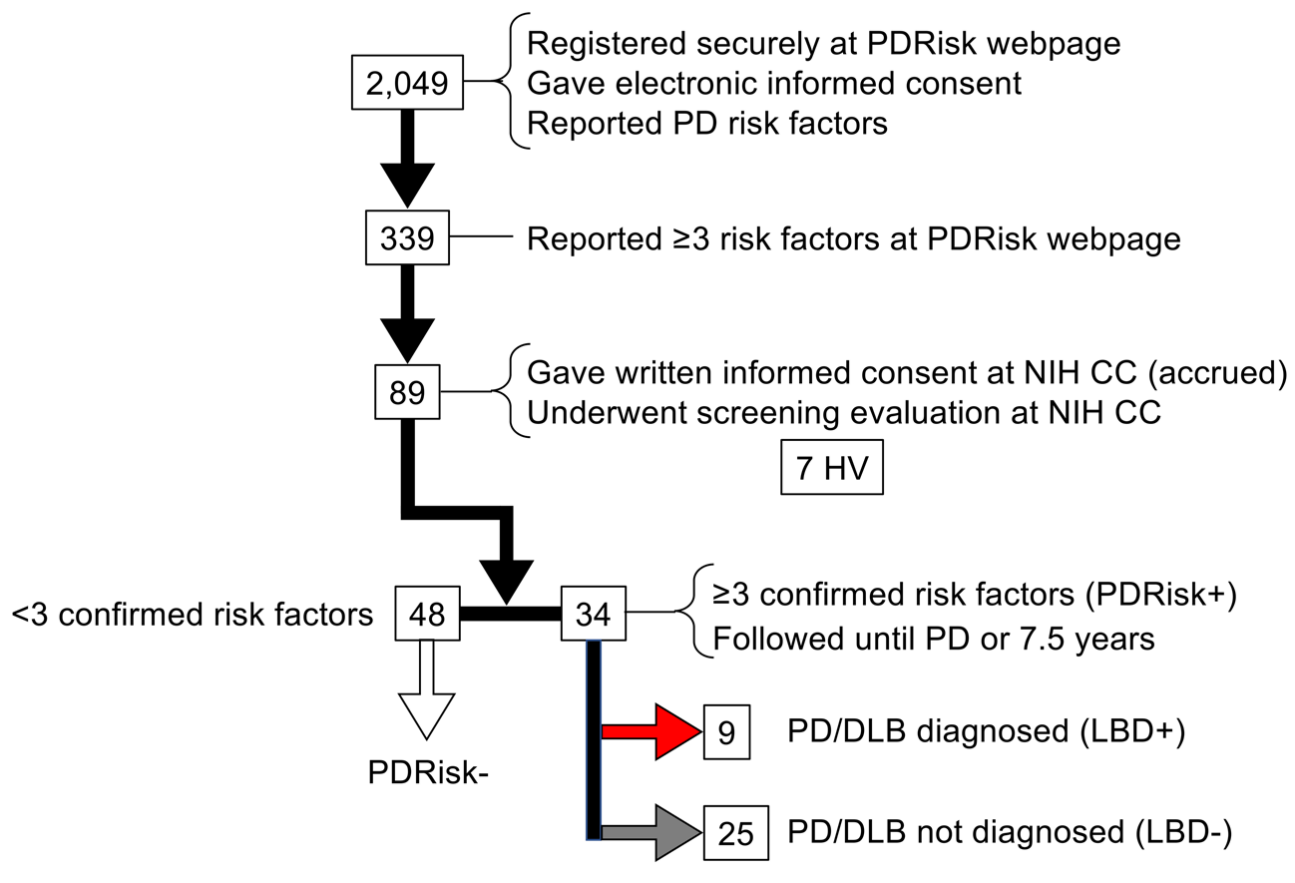
Participants in the PDRisk study. DLB, dementia with Lewy bodies; HV, healthy volunteer; LBD, central Lewy body disease; NIH CC, National Institutes of Health Clinical Center; PD, Parkinson disease; PDRisk, participant in the PDRisk study; PDRisk–, accrued participant in the PDRisk study but without confirmation of ≥3 risk factors upon on-site screening.

Data collection in the PDRisk study began in 2009 and ended in January 2023, after the last follow-up visit of the last participant. A total of 2,094 individuals registered for the study and provided their risk factor information at the protocol-specific website (Figure 1). Of these, 339 (16%) reported 3 or more risk factors and were considered eligible for further participation. After phone interviews of eligible candidates by a research nurse, 82 at-risk individuals came to the NIH Clinical Center for on-site screening, gave written informed consent, and were considered accrued (PDRisk cohort). One at-risk participant was withdrawn during the first admission because of a previously undisclosed exclusion criterion.

### Participant groups.

Thirty-four of the accrued participants in the PDRisk study had confirmation of 3 or more risk factors (PDRisk+) at the time of on-site screening (Table 1); 48 accrued participants did not (PDRisk–) and were not followed as inpatients (Figure 1 and Supplemental Table 1; supplemental material available online with this article; https://doi.org/10.1172/JCI172460DS1).

Included in the PDRisk+ group were 2 individuals who had been tested under another protocol and satisfied the eligibility criteria and consented to follow-up under the PDRisk protocol. For the purposes of data analysis, the follow-up period for these participants began with their first evaluation at the NIH.

Of the 34 accrued participants with 3 or more confirmed risk factors, 9 (26%) subsequently developed a central LBD (LBD+ group; 6 with PD, 2 DLB, and 1 PD+ dementia). One participant was diagnosed with PD by the neurologist in a blinded manner; however, the patient’s death certificate showed she had died of multiple system atrophy (MSA), which is not an LBD, and she was assigned to the LBD– group (LBD– no. 20). This case is described later in detail.

The LBD+ and LBD– groups had similar mean total numbers of risk factors and did not differ in the numbers with a positive family history of PD, olfactory dysfunction, dream enactment behavior, or symptoms of orthostatic intolerance (Table 1 and Supplemental Table 1). The LBD+ and LBD– groups also did not differ in sex or age at study entry or in prescribed medications (Supplemental Table 1). Median follow-up was 2.8 years (mean = 4.7) in the LBD+ group and 6.3 years (mean = 5.1) in the LBD– group. In the LBD+ group, the percentages of participants with positive genetic, olfactory, dream enactment, and orthostatic intolerance risk factors were 89%, 89%, 100%, and 56%, and the corresponding percentages in the LBD– group were 92%, 76%, 80%, and 76%, respectively. The 2 groups did not differ in the frequencies of any of the risk factors.

Data from 11 concurrently studied healthy volunteers (HVs) were included. Of the 11 HVs, 8 had neuroimaging data. Seven HVs were accrued under the PDRisk protocol and the remainder under other IRB-approved protocols.

### Incidence of diagnosed central LBDs in groups stratified by initial ^^^18^^^F-dopamine–derived radioactivity.

Nine PDRisk study participants had low initial myocardial ^^^18^^^F-dopamine–derived radioactivity (<6,000 nCi-kg/cc-mCi) and 25 had normal radioactivity. In the group with low initial radioactivity the median duration of follow-up was 8.1 years (mean = 5.4), and in the group with normal radioactivity the median duration of follow-up was 6.1 years (mean = 4.9). We conducted statistical testing for low versus normal ^^^18^^^F-dopamine–derived radioactivity in groups with complete follow-up data across 7 years of follow-up. Eight of 9 participants with low and 1 of 11 with normal radioactivity subsequently developed a central LBD (Fisher’s exact test *P* = 0.0009).

**Figure 2:**
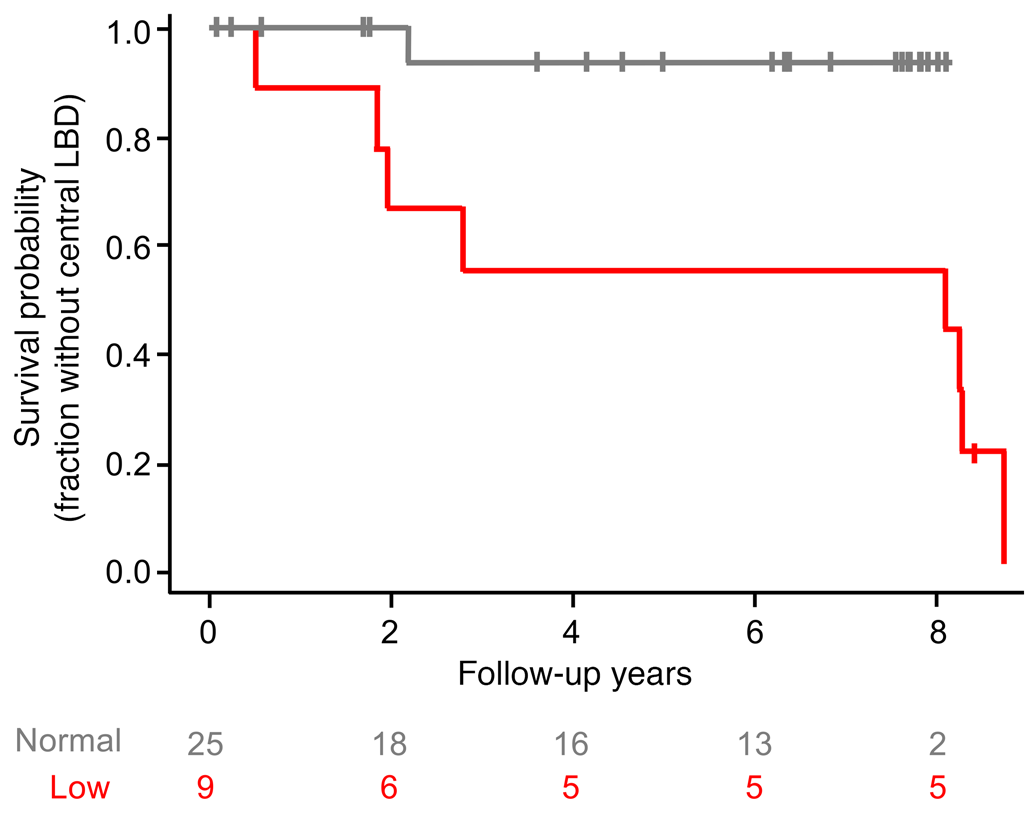
Survival probability (fraction without a central LBD) as a function of years of follow-up in at-risk individuals stratified in terms of the initial interventricular septum ^18^F-dopamine–derived radioactivity. The difference in survival time (without LBD) between low and normal ^18^F-dopamine–derived radioactivity was significant, with *P* = 0.0091 by log-rank test. The bottom 2 lines represent the numbers of participants remaining at risk with normal (>6,000 nCi-kg/cc-mCi) or low (<6,000 nCi-kg/cc-mCi) radioactivity.

Kaplan-Meier curves generated for the dichotomized initial ^^^18^^^F-dopamine–derived radioactivity in the 8-minute dynamic PET frame showed that throughout the period of follow-up the fraction of participants with low radioactivity who developed a central LBD was greater than that of participants with normal radioactivity (log-rank *P* = 0.0091; Figure 2).

The cutoff value of 6,000 nCi-kg/cc-mCi, chosen based on the first-look report (24), used for the survival analysis and Fisher’s exact test. For the data in Table 2, the sensitivity and specificity estimates were determined by receiver operating characteristic (ROC) curve analysis. The cutoff values were similar by the ante hoc and post hoc approaches (6,000 vs. 6,633 nCi-kg/cc-mCi).

It was noted that there were a substantial number of dropouts in the group with normal ^^^18^^^F-dopamine–derived radioactivity, whereas among the individuals with low ^^^18^^^F-dopamine–derived radioactivity none dropped out of the study. Therefore, Kaplan-Meier plots were generated for different periods of follow-up, with *P* values based on the log-rank test. When the follow-up period was less than 4.5 years, the *P* value was 0.0429, and when the follow-up period was less than 5 years, the *P* value was 0.0063 (Supplemental Figure 1).

### Initial cardiac ^^^18^^^F-dopamine–derived radioactivity in the LBD+ vs. LBD– groups.

Nine participants were diagnosed with a central LBD during follow-up (LBD+ group). Of these, 8 (89%) had low initial ^^^18^^^F-dopamine–derived radioactivity. There were 25 participants who were not diagnosed with a central LBD (LBD– group); of these, 1 (4%) had low initial radioactivity (*P* < 0.0001 by Fisher’s exact test).

**Figure 3:**
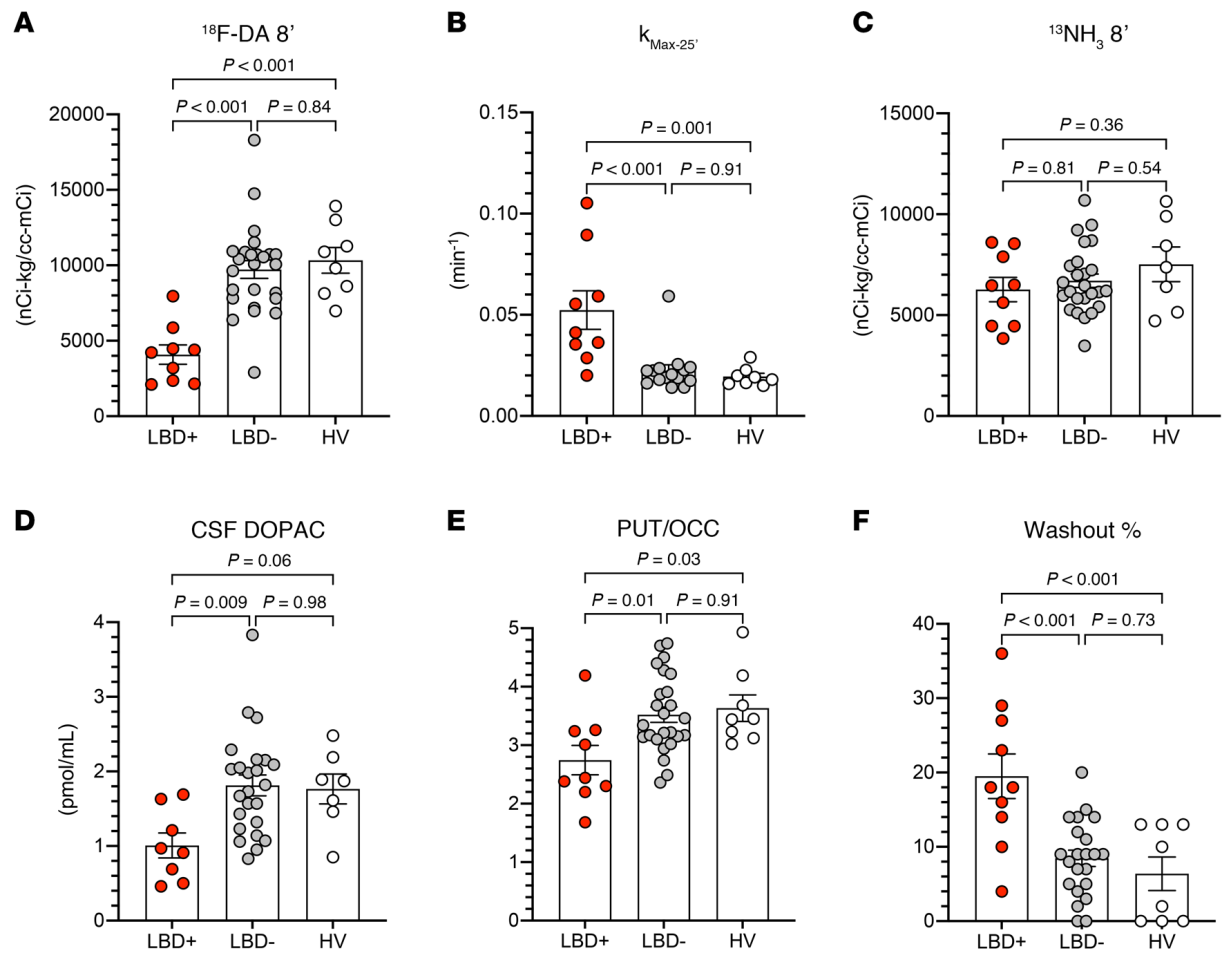
Individual and mean (±SEM) values for cardiac PET data and biomarkers of central dopaminergic innervation in at-risk individuals who subsequently were diagnosed with a central Lewy body disease (LBD+) or were not diagnosed with a central Lewy body disease (LBD–) and concurrently studied healthy volunteers (HVs). (**A**) ^18^F-dopamine–derived (^18^F-DA–derived) radioactivity in the dynamic PET frame with the midpoint 8 minutes after initiation of intravenous administration of the tracer. (**B**) Mono-exponential slope of decline in ^18^F-DA–derived radioactivity across the time points from the maximum value to the value in the dynamic frame with the midpoint 25 minutes after initiation of infusion of the tracer (*k*_Max–25′_). (**C**) ^13^N-ammonia–derived (^13^NH_3_-derived) radioactivity in the dynamic PET frame with the midpoint 8 minutes after initiation of intravenous administration of the tracer. (**D**) Cerebrospinal fluid concentration of 3,4-dihydroxyphenylacetic acid (DOPAC). (**E**) Putamen/occipital (PUT/OCC) cortex ratios of ^18^F-DOPA–derived radioactivity. (**F**) Percentage decrease in putamen ^18^F-DOPA–derived radioactivity between the 15-minute static scan beginning 30 minutes after intravenous administration of the tracer and the 15-minute static scan ending 120 minutes after administration of the tracer. Statistical testing was 1-way ANOVA with Tukey’s post hoc test for multiple comparisons.

ANOVAs with Tukey’s post hoc test showed that for 6 biomarkers of ^^^18^^^F-dopamine–derived radioactivity the differences between the LBD+ and the LBD– and HV groups were significant, whereas the HV and LBD– groups did not differ (Table 1). Age was used as a covariate, while sex was not associated with any outcomes and was dropped as a covariate. The LBD+ group had lower mean 8-minute ^^^18^^^F-dopamine–derived radioactivity (Figure 3A) and higher mean *k*___Max–25′___ (where 25′ is 25 minutes; Figure 3B) than did the LBD– group.

**Figure 4:**
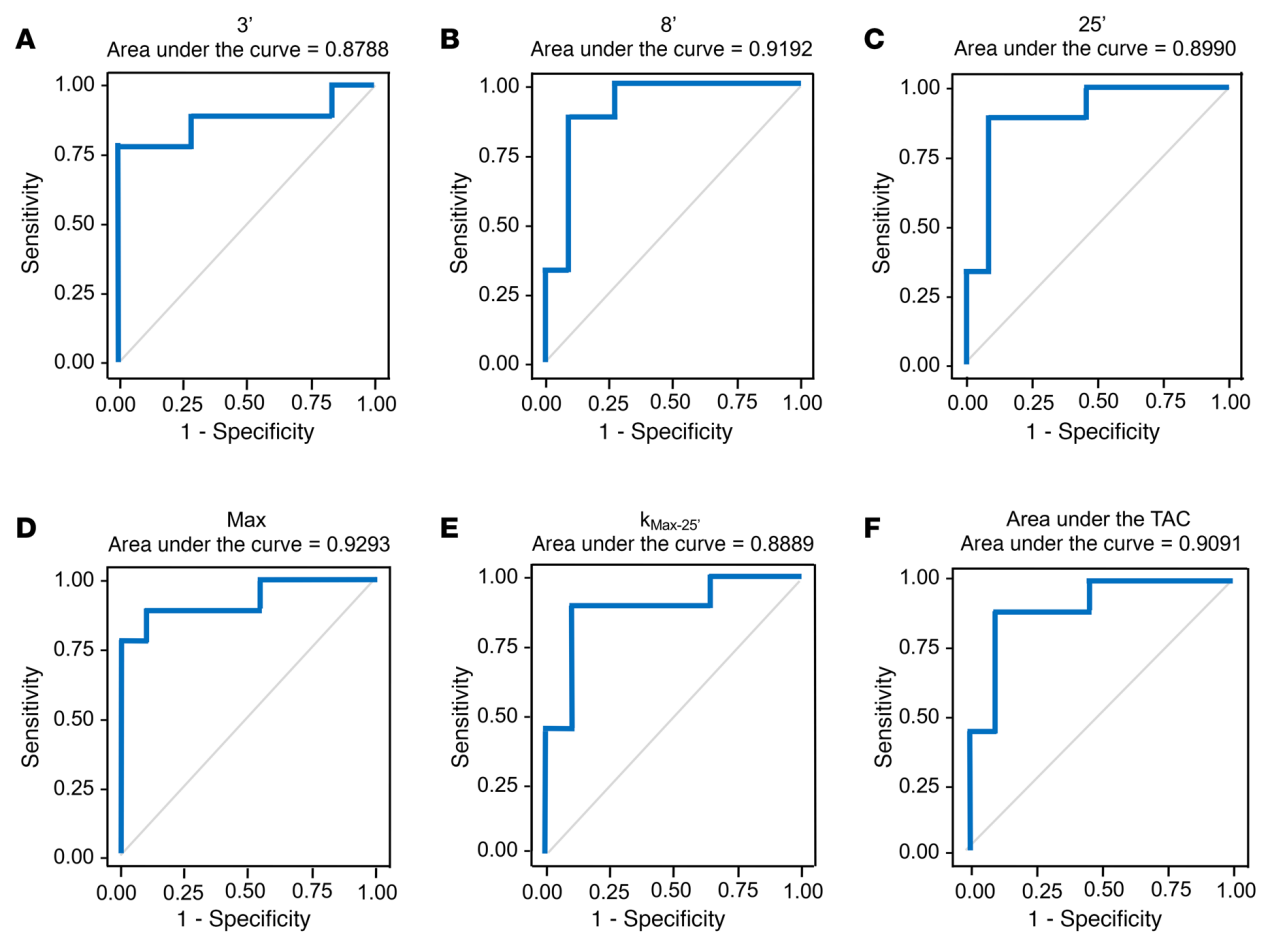
Receiver operating characteristic curves for interventricular septal myocardial ^18^F-dopamine–derived radioactivity in participants with ≥7 years follow-up (9 LBD+, 11 LBD–). (**A**) ^18^F-dopamine–derived (^8^F-DA–derived) radioactivity in the dynamic PET frame with the midpoint 3 minutes after initiation of intravenous administration of the tracer. (**B**) ^18^F-DA–derived radioactivity in the dynamic PET frame with the midpoint 8 minutes after initiation of intravenous administration of the tracer. (**C**) ^18^F-DA–derived radioactivity in the dynamic PET frame with the midpoint 25 minutes after initiation of intravenous administration of the tracer. (**D**) Maximum ^18^F-DA–derived radioactivity among the dynamic PET frames. (**E**) Mono-exponential slope of decline in ^18^F-DA–derived radioactivity across the time points from the maximum value to the value in the dynamic frame with the midpoint 25 minutes after initiation of infusion of the tracer (*k*_Max–25′_). (**F**) Area under the curve for ^18^F-DA–derived radioactivity across the dynamic PET frames.

ROC curves were constructed to evaluate the measures of ^^^18^^^F-dopamine–derived radioactivity for their efficiency in separating the LBD+ from LBD– groups (Figure 4 and Table 2). Based on the areas under the ROC curves (AUCs), all 6 biomarkers showed a high degree of separability (AUC ≥ 0.85). Sensitivities and specificities based on Youden’s method showed high values for the 6 biomarkers, comparing the LBD+ group versus the LBD– subgroup with 7 or more years of follow-up and versus the entire LBD– group (Table 3).

The mean initial plasma norepinephrine concentration in the LBD+ group was 1.16 ± 0.15 pmol/mL, the LBD– group 1.91 ± 0.17, and the PDRisk– group 2.07 ± 0.21 pmol/mL. The corresponding mean initial 3,4-dihydroxyphenylglycol (DHPG) concentrations were 4.46 ± 0.63, 5.90 ± 0.34, and 5.89 ± 0.40 pmol/mL, respectively. The groups did not differ in mean values for levels of either analyte.

In most of the 25 participants with 3 or more risk factors in the LBD– group, further follow-up information was not obtained after reaching the study endpoint. Two of the PDRisk study participants phenoconverted to a central LBD during the COVID-19 pandemic. Review of their clinical history information excluded post–COVID-19 syndrome.

Some evaluations were delayed during the follow-up period, especially due to the COVID-19 pandemic. One participant developed DLB after he had completed the PDRisk study and was included in the LBD+ group.

### Trends in cardiac ^^^18^^^F-dopamine–derived radioactivity during follow-up in the LBD+ and LBD– groups.

**Figure 5:**
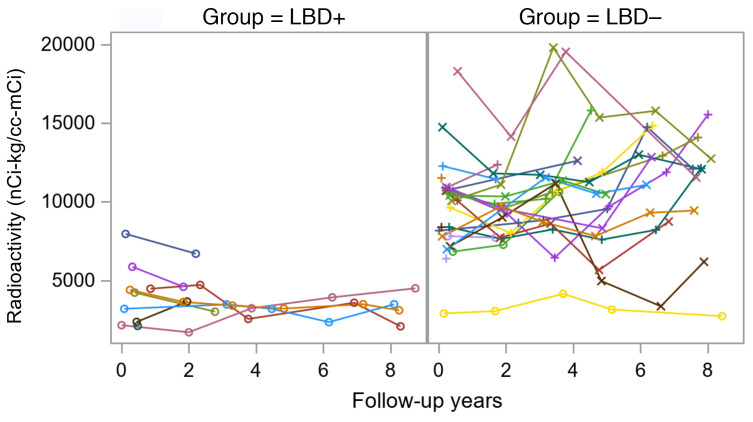
Myocardial ^18^F-dopamine–derived radioactivity during follow-up. Individual observed values for interventricular septal myocardial ^18^F-dopamine–derived radioactivity over of years of follow-up in at-risk individuals who subsequently were diagnosed with a central Lewy body disease (LBD+, left) or were not diagnosed with a central Lewy body disease (LBD–, right) during follow-up. Each colored line shows data for 1 participant. Left: Individual values for ^18^F-dopamine–derived radioactivity in the 8-minute dynamic frame in the LBD+ group. Right: Individual values for ^18^F-dopamine–derived radioactivity in the 8-minute dynamic frame in the LBD– group.

In both the LBD+ and LBD– groups, cardiac ^^^18^^^F-dopamine–derived radioactivity was stable over years of follow-up (Figure 5).

### Comparisons of kinetics of ^^^18^^^F-dopamine–derived radioactivity within PET sessions in LBD+, LBD–, and HV groups.

**Figure 6:**
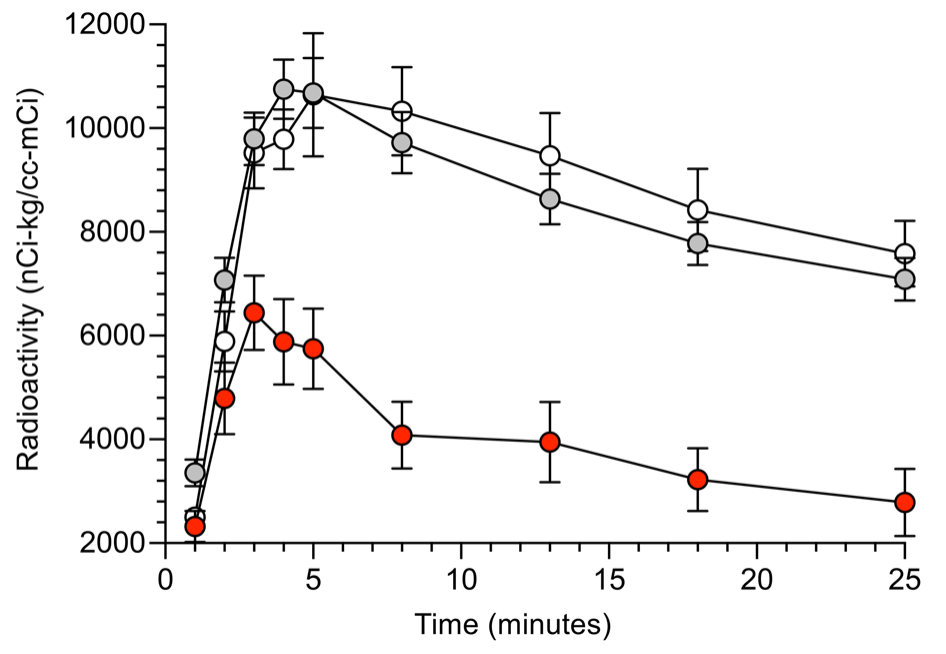
Myocardial ^18^F-dopamine–derived radioactivity during the initial positron emission tomography session. Mean (±SEM) values for interventricular septal myocardial ^18^F-dopamine–derived radioactivity in at-risk individuals who subsequently were diagnosed with a central Lewy body disease (LBD+, red, *n* = 9) or were not diagnosed with a central Lewy body disease (LBD–, gray, *n* = 25) and in concurrently studied healthy volunteers (HVs, white, *n* = 8). The LBD+ group had lower mean ^18^F-dopamine–derived radioactivity than the LBD– and HV groups at all time points after infusion of the tracer.

In all participants, cardiac ^^^18^^^F-dopamine–derived radioactivity increased rapidly during the 3-minute administration of the tracer (Figure 6 and Table 4). The LBD+ and LBD– groups differed in septal ^^^18^^^F-dopamine–derived radioactivity at all time points after the 3-minute administration of the tracer, whereas the LBD– and HV groups did not differ at any time point. The mono-exponential slope of decline in radioactivity between the peak value and 25 minutes, *k*___Max–25′___, was greater in the LBD+ than the LBD– group (*P* < 0.001; Figure 3B).

**Figure 7:**
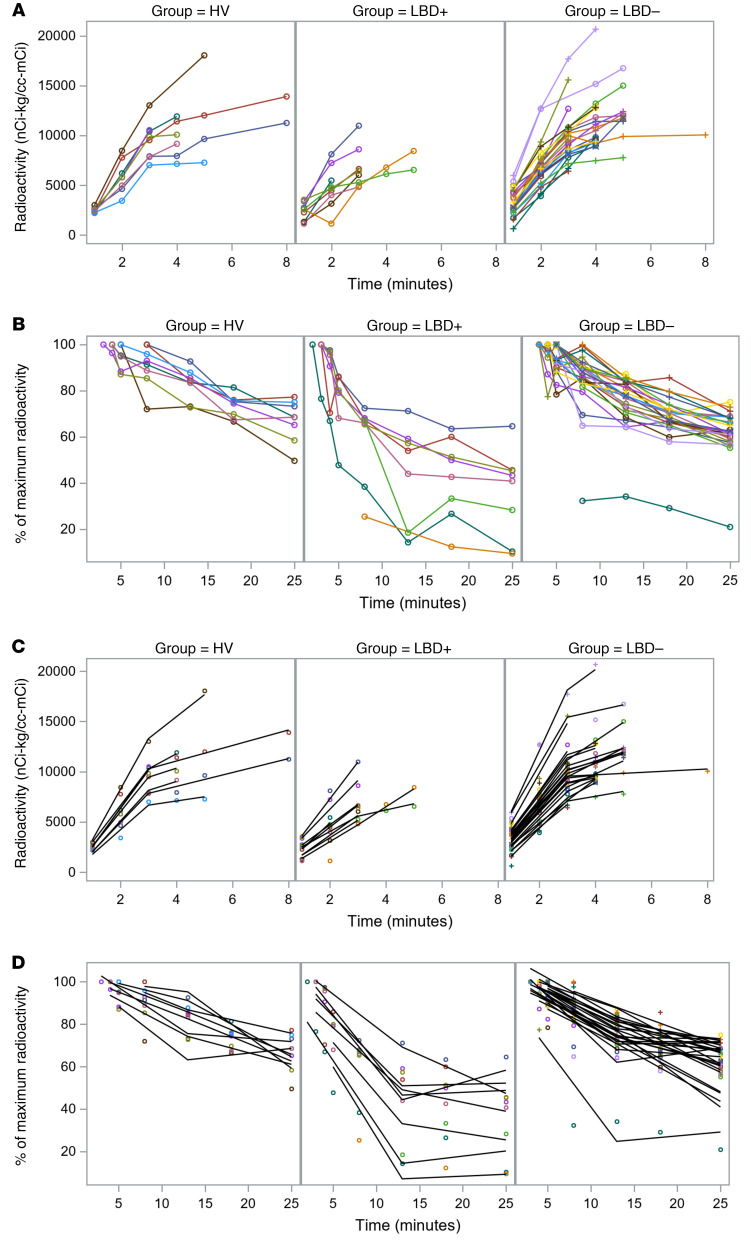
Modeling uptake and subsequent loss of ^18^F-dopamine–derived radioactivity. Individual observed values (**A** and **B**) and predicted curves of best fit (**C** and **D**) for interventricular septal myocardial ^18^F-dopamine–derived radioactivity as a function of time from initiation of 3-minute intravenous administration of the tracer in healthy volunteers (HV) and in at-risk individuals subsequently diagnosed with a central Lewy body disease (LBD+) or not diagnosed with a central Lewy body disease during follow-up (LBD–). (**A** and **C**) Data from 1 minute after initiation of 3-minute intravenous administration of the tracer to the maximum radioactivity. (**B** and **D**) Data from maximum radioactivity to 25 minutes after initiation of administration of the tracer, expressed as percentage of maximum radioactivity. In **A** and **C**, the early increase in radioactivity was slower in the LBD+ than LBD– and HV groups. In **B** and **D**, the LBD+ group had more rapid loss of radioactivity than the LBD– and HV groups between the maximum radioactivity and 13 minutes. The outlier data at 3 minutes for patient LBD– no. 11 and the data at 4 minutes for HV no. 4 were excluded from the analysis. From peak to 25 minutes the excluded outlier time point data are at 5 and 13 minutes for LBD+ no. 9, 5 minutes for LBD+ no. 10, 4 and 5 minutes for LBD– no. 3, and peak to 25 minutes for LBD+ no. 4.

The results of the random coefficient model analysis for time equal to 1 minute to the time of peak radioactivity revealed decreased uptake of ^^^18^^^F-dopamine in the LBD+ group (Table 4 and Figure 7). The slope for time equal to 3 minutes to maximum radioactivity was not significant for the LBD+ group but was for the other 2 groups. For each group, the difference in slope between time ≤3 minutes and time ≥3 minutes was significant, and the increase (slope > 0 nCi-kg/cc-mCi per minute) for time ≤3 minutes was faster than after time ≥3 minutes. For time ≤3 minutes, the difference in slope between the LBD+ and the HV and LBD– groups was significant.

The random coefficient model analyses for time equal to peak time to 25 minutes showed more rapid proportionate loss of ^^^18^^^F-dopamine–derived radioactivity in the LBD+ group between the peak radioactivity and 13 minutes, but not between 13 and 25 minutes. In the period from peak time to 13 minutes, the decline in radioactivity was significantly faster in the LBD+ group than in the HV and LBD– groups (*P* < 0.001; Table 4 and Figure 7, B and D). In the period of time from 13 to 25 minutes, the slope of decline in radioactivity in the LBD+ group was significantly slower than in the HV and LBD– groups (*P* < 0.0001).

### Correlations among biomarkers of cardiac noradrenergic deficiency.

The 6 biomarkers of ^^^18^^^F-dopamine–derived radioactivity were significantly correlated (Supplemental Table 2). Correlation coefficients between *k*___Max–25′___ and the other 5 biomarkers were moderately negatively correlated, with the extent of correlation related to the time of the dynamic frame (relatively high Pearson’s *r* values for the 8-, 13-, 18-, and 25-minute frames (*r* = –0.63, –0.56, –0.67, and –0.70, respectively; *P* < 0.0001 for each).

### Biomarkers of central dopaminergic innervation and relationships to biomarkers of cardiac noradrenergic innervation.

The LBD+ group had lower mean CSF levels of DOPAC, lower putamen/occipital cortex (PUT/OCC) ratios of ^^^18^^^F-DOPA–derived radioactivity, and greater putamen washout percentages of ^^^18^^^F-DOPA–derived radioactivity than did the LBD– group (Figure 3, D–F).

**Figure 8:**
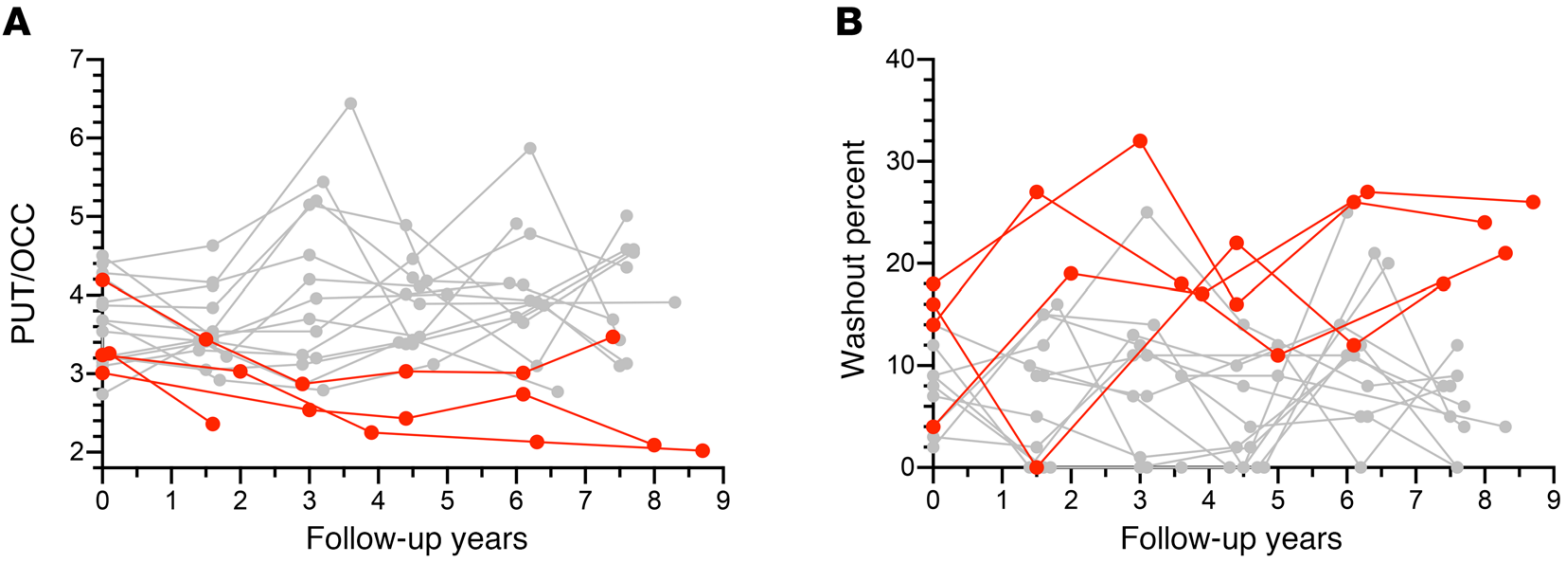
^18^F-DOPA–derived radioactivity during follow-up. Putamen/occipital cortex ratios (PUT/OCC) (**A**) and washout percentages of putamen ^18^F-DOPA–derived radioactivity (**B**) in participants developing a central Lewy body disease during follow-up (LBD+, red) and in participants not developing a central LBD after at least 4 years of follow-up (LBD–). (**A**) LBD+ participants who initially had normal PUT/OCC ratios (>2.7). (**B**) LBD+ participants who initially had normal washout percentages (<20%). PUT/OCC ratios decreased and washout percentages increased between the time of the initial evaluation at the time of diagnosis with a central LBD.

Among the LBD+ participants, in 4 the PUT/OCC ratio of ^^^18^^^F-DOPA–derived radioactivity initially was above the cutoff value of 2.7 (Figure 8). All 4 had a decrease in their PUT/OCC ratios between the initial evaluation and the time of diagnosis of a central LBD. Four individuals in the LBD+ group had initial washout percentages of putamen ^^^18^^^F-DOPA–derived radioactivity that were below the cutoff value of 20%. All 4 had increases in washout percentages between the time of initial evaluation and the time of diagnosis of a central LBD. Therefore, in a substantial minority of the LBD+ group, low cardiac ^^^18^^^F-dopamine–derived radioactivity preceded low putamen ^^^18^^^F-DOPA–derived radioactivity.

Across the LBD+, LBD–, and HV groups individual values for washout percentages of putamen ^^^18^^^F-DOPA–derived radioactivity were negatively correlated with PUT/OCC ratios and CSF DOPAC levels, while PUT/OCC ratios were unrelated to CSF DOPAC levels (Supplemental Table 2). Individual values for the washout percentages of putamen ^^^18^^^F-DOPA–derived radioactivity were unrelated to values for cardiac *k*___Max–25′___.

### Myocardial perfusion.

Myocardial perfusion as indicated by interventricular septal myocardial ^^^13^^^N-ammonia–derived radioactivity did not distinguish the LBD+ from the LBD– and HV groups (Figure 3C).

### Autonomic function tests.

Supplemental Table 1 (Physiological Initial tab) summarizes data from autonomic function testing upon initial evaluation in the LBD+ and LBD– groups. Data from the LBD– patient with MSA were excluded from the statistical analyses. The LBD+ group had lower mean heart rates during supine rest (*P* = 0.00439) and head-up tilt (*P* = 0.02036) and lower blood pressures during tilt, but the groups did not differ in blood pressure during supine rest.

The LBD+ group had lower initial mean values for indices of baroreflex-sympathoneural function — the log of the baroreflex area in phase III of the Valsalva maneuver (*P* = 0.00721) and the log of the pressure recovery time (*P* = 0.00467). The groups did not differ in mean values for indices of baroreflex-cardiovagal function — the baroslope during phase II of the Valsalva maneuver (43) and ΔHR/ΔBPs (44) during head-up tilt. The groups did not differ in indices of heart rate variability in either the time or frequency domain.

## Discussion

The results of this prospective, observational, long-term study highlight the ability of ^^^18^^^F-dopamine PET to identify preclinical central LBDs (PD or DLB) in at-risk individuals. The main finding was that among individuals with multiple PD risk factors, low cardiac ^^^18^^^F-dopamine–derived radioactivity highly efficiently predicted the subsequent diagnosis of a central LBD.

In the overall PDRisk cohort of individuals with 3 or more risk factors, 26% were diagnosed with a central LBD during follow-up of up to about 7.5 years, a larger percentage than would be expected in the general population (45, 46). These data confirm the potency of the chosen combination of risk factors. Low ^^^18^^^F-dopamine–derived radioactivity increased to a virtual certainty the likelihood of developing a central LBD. Meanwhile, neuroimaging evidence of intact cardiac noradrenergic innervation decreased to near zero the likelihood of a subsequent central LBD, despite the same risk factors. Shorter follow-up time in the LBD+ group would be expected, because the follow-up ended at the time of a diagnosis of PD.

### Persistent abnormalities of cardiac sympathetic innervation in preclinical central LBDs.

There were no temporal trends in radioactivity in either the LBD+ or LBD– group. Almost all the LBD+ participants already had low radioactivity upon initial evaluation, and almost all the LBD– participants had normal radioactivity throughout the period of follow-up. Cardiac noradrenergic deficiency probably was already advanced in most of the LBD+ group, even upon initial evaluation.

The data do not address the time course for development of cardiac noradrenergic deficiency, with one exception. In one LBD+ participant (LBD+ no. 1), initial interventricular septal cardiac ^^^18^^^F-dopamine–derived radioactivity was normal, although apical radioactivity was decreased. Approximately 2 years later, when the participant was diagnosed with PD, apical and free-wall radioactivity both were decreased, and the time-activity curve for septal radioactivity was shifted downward, a pattern typical of progression of cardiac sympathetic denervation in PD without OH (47). Counting this individual, all the at-risk participants in the PDRisk study who were diagnosed with a central LBD during follow-up had evidence of antecedent cardiac noradrenergic deficiency by ^^^18^^^F-dopamine PET.

### When does cardiac noradrenergic deficiency occur with respect to central dopaminergic deficiency in central LBDs?

Among the 9 participants in the LBD+ group, 4 had normal initial PUT/OCC ratios of ^^^18^^^F-DOPA–derived radioactivity, and 4 had normal initial washout percentages of putamen ^^^18^^^F-DOPA–derived radioactivity. During follow-up, the PUT/OCC ratios decreased and washout percentages increased, whereas there were no trends in either biomarker in the LBD– group. In 3 of the 4 LBD+ individuals with normal initial PUT/OCC ratios, by the time of diagnosis the ratios were below the cutoff value, and in 3 of the 4 LBD+ individuals with normal initial washout percentages, by the time of diagnosis the washout percentages were above the cutoff value.

Since upon initial evaluation all the participants in the LBD+ group had evidence of diffuse or localized noradrenergic deficiency in the left ventricular myocardium, the data indicate that the LBD+ group included a substantial subgroup in whom cardiac noradrenergic deficiency preceded putamen dopamine deficiency, consistent with previous case reports of PD (9) or DLB (10).

### Combination of denervation with a vesicular storage defect in preclinical central LBDs.

A particular functional abnormality in residual cardiac sympathetic nerves — inefficient vesicular sequestration of catecholamines — was associated with an increased probability of subsequently developing a central LBD. The ROC curve AUC of 0.92 for *k*___Max–25′___ in the LBD+ and LBD– groups indicated excellent ability of this functional biomarker for distinguishing the 2 groups (sensitivity 89% at specificity 96%). Denervation alone cannot explain accelerated loss of ^^^18^^^F-dopamine–derived radioactivity (48). Decreased uptake of ^^^18^^^F-dopamine in the LBD+ group could not be attributed to decreased coronary perfusion, as the LBD+ and LBD– groups had similar time-activity curves for ^^^13^^^N-ammonia–derived radioactivity.

The breakpoint analyses showed that individuals with preclinical central LBDs had at least some degree of cardiac sympathetic denervation. If there were only a vesicular storage defect, then the increase in radioactivity between the 3-minute and peak radioactivity would be attenuated in the LBD+ group, and this was not the case. The LBD+ group also had at least some degree of a vesicular storage defect, because if there were only a loss of sympathetic nerves, then the rate of decrease in radioactivity from the peak value to 13 minutes would be normal, and this also was not the case. We therefore infer that low cardiac ^^^18^^^F-dopamine–derived radioactivity in the LBD+ group reflected a combination of denervation with a vesicular storage defect in residual sympathetic nerves, as predicted by computational modeling applied to intraneuronal determinants of norepinephrine stores in cardiac sympathetic nerves in LBDs (5).

The LBD+ group also had increased washout percentages of putamen ^^^18^^^F-DOPA–derived radioactivity, consistent with the view that decreased vesicular storage in catecholaminergic neurons occurs in the nigrostriatal dopaminergic system (49). Postmortem neurochemical analysis has confirmed the occurrence of a vesicular storage defect in the putamen in LBDs (50). This has also been demonstrated directly in isolated striatal vesicles from patients with PD (42).

### Pure autonomic failure may be a prototype of body-first central LBDs.

Two of the LBD+ participants had pure autonomic failure (PAF), a rare disease characterized by neurogenic OH, generalized sympathetic noradrenergic deficiency, and intraneuronal deposition of αS in skin biopsies (51), without clinical evidence of motor or cognitive impairment. PAF can go on for many years without symptoms or signs of a central LBD (52), but PAF can evolve to PD or DLB (9, 10, 23, 53), and postmortem neuropathology has documented brainstem Lewy bodies in virtually all PAF patients (53).

In 1 PAF patient in the LBD+ group (LBD no. 8), PUT/OCC ratios of ^^^18^^^F-DOPA–derived radioactivity showed a triphasic downward trend over the years of follow-up, mirroring the triphasic pattern found in cardiac norepinephrine stores in LBDs (35). A transition from normal to rapid decline in PUT/OCC ratios might identify the optimal time for initiation of treatment. Borghammer and colleagues have proposed “brain-first” and “body-first” pathophysiological sequences in PD (14). The choice of risk factors for entry into the PDRisk study probably is biased toward the body-first sequence. One of the participants in the LBD+ group initially had normal interventricular septal ^^^18^^^F-dopamine–derived radioactivity, which might fit with the brain-first sequence; however, this participant also had initially normal putamen ^^^18^^^F-DOPA–derived radioactivity, and values for both variables became abnormal at about the same time that he was diagnosed with PD. Perhaps there is a third possible pathogenetic sequence besides brain-first versus body-first. If the inciting agent were both inhaled and swallowed, then early brain and autonomic involvement could occur approximately simultaneously.

### Central and peripheral nonmotor aspects of LBDs are correlated with each other.

RBD is a strong risk factor for subsequent development of a neurodegenerative synucleinopathy (54), including a “diffuse/malignant” form of PD (55) that features OH (56, 57). RBD is also associated with olfactory dysfunction (58, 59) and neuroimaging evidence of cardiac noradrenergic deficiency (60). Longitudinal studies of RBD patients have led to the view that autonomic dysfunction can occur early in neurodegenerative synucleinopathies, years before the motor or cognitive onset of central LBDs (22). The present data fit with substantial overlaps among dream enactment behavior, olfactory dysfunction, OH, and cardiac noradrenergic deficiency in preclinical central LBDs.

### Evolution to MSA.

One PDRisk study participant with neurogenic OH (LBD– no. 20) had persistently normal ^^^18^^^F-dopamine–derived radioactivity and normal scores on the University of Pennsylvania Smell Identification test (UPSIT), findings that argue strongly against PD+OH (49, 61). The patient was diagnosed with PD by the blinded neurologist. A few years after reaching the study endpoint, the patient died. According to the official Certificate of Death, the cause of death was MSA. Even an expert movement-disorders neurologist may not be able to distinguish PD+OH from the parkinsonian form of MSA by clinical examination alone; however, consistently normal ^^^18^^^F-dopamine–derived radioactivity rules out PD+OH (62).

Where in the autonomic nervous system do central LBDs begin? The present results support the view that the pathogenetic process leading to central LBDs often begins outside the brain, in the autonomic nervous system. How synucleinopathy in the enteric nervous system, in vagal nerve fibers, in myocardium, and in sympathetic noradrenergic axons in skin biopsies (63) are related remains poorly understood.

### Study limitations.

The PDRisk study involved a relatively small number of participants. This was because of the strong hypothesis and the power calculation when the study was designed. We believe that the fact that the results from applying different testing approaches agreed with each other reinforces the validity of the conclusions by convergence (consilience) (64).

Since all the PDRisk participants had multiple risk factors, generalizability to people with fewer or different risk factors could not be determined.

The protocol did not specify the criteria (e.g., those promulgated by the Movement Disorders Society or UK Brain Bank) that would be used by the board-certified, blinded neurologist to diagnose PD.

We debriefed all the participants about their PET results at each follow-up visit. This might explain the high rate of dropouts in the group with normal ^^^18^^^F-dopamine–derived radioactivity, since they may have thought they would not develop PD, whereas individuals with low radioactivity continued participation out of concern that they would develop PD.

^^^18^^^F-dopamine PET is only available at the NIH Clinical Center. We hope that the results reported here documenting the ability to identify preclinical central LBDs will induce researchers at other institutions to join the Investigational New Drug (IND) application for ^^^18^^^F-dopamine (IND 33,866) or apply for an IND, about which we would be happy to advise or collaborate. This technology should be applied in larger populations of less stringently selected at-risk individuals, a type of study that cannot be done solely within the NIH. ^^^123^^^I-metaiodobenzylguanidine (^^^123^^^I-MIBG) single photon emission computed tomography (SPECT) is available at most centers for diagnostic evaluation of pheochromocytoma, but insurance carriers in the United States do not cover ^^^123^^^I-MIBG SPECT in known or suspected LBDs. We have commented about this for many years (65, 66). There are theoretical advantages of ^^^18^^^F-dopamine PET over ^^^123^^^I-MIBG SPECT (67). Particularly relevant to the present report, ^^^123^^^I-MIBG SPECT cannot separate denervation from a vesicular storage defect in residual sympathetic nerves.

### Implications.

The present results have clear implications for treatment and prevention trials for individuals at high risk of central LBDs. Biomarkers of cardiac noradrenergic deficiency seem to identify a disease process that will progress to a central LBD. Assessing these biomarkers in at-risk individuals may be valuable for efficient selection of eligible candidates and for assessing effects of potential neurorescue or neuroprotective approaches objectively and quantitatively. Computational modeling indicates that strategies targeting toxic catecholaldehyde-αS interactions in catecholaminergic neurons would substantially delay the onset of symptomatic LBDs (35). We propose that a clinical trial involving a relatively small number of highly selected participants should enable testing for the first time of whether central LBDs can be prevented in at-risk individuals.

## Methods

Further details can be found in the Supplemental Methods.

### Recruitment.

Recruitment into the study was via an IRB-approved advertisement directing visitors to an IRB-approved, protocol-specific, secure website. Those interested in registering for the study received confidential, unique identification numbers via email. After consenting to the study electronically in compliance with the Privacy Rule (45 CFR 164.501, 164.508, 164.512[i]), participants were queried about 4 types of risk factor — genetic, olfactory, dream enactment behavior, and OH or orthostatic intolerance. Candidate participants who reported at least 3 risk factors on the website were interviewed by phone to confirm the self-reported information and select participants for on-site screening.

### Inclusion and exclusion criteria.

Eligibility criteria for the PDRisk study included a participant age of 18 years old or older. A candidate participant was excluded if there were any disqualifying medical conditions (e.g., insulin-dependent diabetes mellitus, symptomatic cerebrovascular or coronary heart disease, renal failure), PD had already been diagnosed, the registrant was more than 70 years old, or clinical considerations required continued treatment with a drug likely to interfere with the scientific results (https://clinicalstudies.info.nih.gov/ProtocolDetails.aspx?id=2009-N-0010#eligibility). Recruitment was done independently of sex, race, ethnicity, or national origin.

### Screening examination.

The purposes of the on-site screening examination at the NIH Clinical Center were to obtain consent for further participation in the study, verify at least 3 risk factors, and perform clinical laboratory tests as described below.

A positive family history (≥1 first-degree relative or ≥2 second-degree relatives with PD) confirmed at the time of on-site screening at the NIH Clinical Center satisfied the genetic criterion. Decreased sense of smell verified by the UPSIT (moderate or severe microsmia or anosmia) (68) satisfied the olfactory criterion. To satisfy the dream enactment risk factor criterion, the individual or a member of the individual’s household must have reported that the individual had movements of the body or limbs associated with dreaming and at least one of the following: potentially harmful sleep behavior, dreams that appeared to be acted out, and sleep behavior that disrupted sleep continuity (69). A polysomnographic diagnosis of RBD was not required. To satisfy the OH risk factor criterion, the individual must have reported symptoms of OH or having OH, corroborated at the screening evaluation.

Participants with 3 or more confirmed risk factors underwent inpatient biomarker testing and follow-up over five 1.5-year intervals (total 7.5 years) or until diagnosed with PD.

If between the time of determination of eligibility and the time of on-site screening PD had been diagnosed, or PD was evident at the time of screening, then the participant was not invited for the inpatient testing and follow-up.

### Outcome measures.

The primary outcome measure was a diagnosis of PD by a neurologist who was blinded as to the results of the biomarker testing. PD was diagnosed according to accepted clinical criteria such as bradykinesia, rigidity, resting tremor, and imbalance. DLB was diagnosed based on cognitive dysfunction, parkinsonism, and 2 other core features — visual hallucinations and fluctuating attention and concentration (70, 71). A diagnosis of DLB was not designed in as an outcome measure.

The clinical diagnosis of PD was left to the blinded neurologist at each follow-up visit. We did not require that the neurologist apply any particular criteria.

### Study sample size.

The PDRisk study had a strong primary hypothesis, which was that among at-risk individuals, positive biomarkers of catecholaminergic neurodegeneration in the heart or brain identify premotor PD. To estimate the required numbers of participants we used a log-rank test for evaluating the association between biomarkers and LBD+ diagnosis, and predicted that among individuals who were at risk of PD and had abnormal catecholaminergic biomarkers, 80% would develop LBD+ by 7.5 years of follow-up; and that among at-risk participants without abnormal biomarkers, 20% would develop LBD+ during follow-up. Power analysis indicated that 26 participants would be sufficient (24, 72) to detect the association at an α value of 0.05 and β value of 0.20.

### Study groups.

The PDRisk study cohort was stratified into 3 groups — a group that during follow-up was diagnosed with a central LBD (LBD+), a group that during follow-up was not diagnosed with a central LBD (LBD–), and a group that upon screening evaluation did not have at least 3 confirmed risk factors and was not invited to return for follow-up testing (PDRisk–). For comparison purposes, a concurrent control group of age-matched HVs was included.

### Dynamic ^^^18^^^F-dopamine PET.

In the PDRisk study, dynamic PET images were obtained in five 1-minute frames, then three 5-minute frames (midpoints about 8, 13, and 18 minutes, and a final 10-minute frame with the midpoint 25 minutes from the time of initiation of the infusion.

The main outcome measurement variable was the amount of ^^^18^^^F-dopamine–derived radioactivity during the 5-minute time frame with the midpoint approximately 8 minutes from initiation of tracer administration, as we have used this variable in several previous studies (24, 35, 49, 62, 73–75). Other outcome measurement variables were the amount of radioactivity in the frame when the infusion ended (midpoint 3 minutes from initiation of tracer administration), radioactivity in the last dynamic frame (midpoint about 25 minutes), maximum radioactivity, the slope of mono-exponential decline in radioactivity from the maximum radioactivity to 25 minutes (*k*___Max–25′___) in units of 1/min and the area under the time-activity curve (Area TAC) for radioactivity across the 9 dynamic frames, calculated based on the trapezoidal rule. The values for Area TAC were standardized by dividing by 25 minutes, the midpoint of the last scanning frame.

The cutoff value for an increased slope of decline in ^^^18^^^F-dopamine–derived radioactivity during a PET scanning session was 0.028/min or higher, the cutoff value for a decreased PUT/OCC ratio of ^^^18^^^F-DOPA–derived radioactivity was less than 2.7, and the cutoff value for an increased washout percentage of putamen ^^^18^^^F-DOPA–derived radioactivity was greater than 20% (48, 49).

### Neurochemical and neuroimaging indices of central dopamine deficiency.

Most of the PDRisk+ participants underwent both lumbar puncture for assays of CSF levels of catechols and brain ^^^18^^^F-DOPA PET, by methods described previously by our group (72). Briefly, lumbar puncture was done under fluoroscopic guidance. One-milliliter aliquots of CSF were frozen immediately on dry ice and kept frozen at –80°C until assayed for catechols by batch alumina extraction followed by liquid chromatography with series electrochemical detection. For ^^^18^^^F-DOPA PET, 7 mCi of the tracer was administered intravenously without carbidopa pretreatment. A static 15-minute emission scan was obtained beginning at 30 minutes after tracer administration, and a static 15-minute emission scan was obtained ending at 120 minutes after tracer administration. The PUT/OCC ratio of ^^^18^^^F-DOPA–derived radioactivity was calculated for the second emission scan (49). The percentage washout of radioactivity was calculated from the PUT radioactivity in the first scan minus the radioactivity in the second scan, the result of which was then divided by the radioactivity in the first scan.

### Autonomic function testing.

Autonomic function testing in the LBD+, LBD–, and PDRisk– groups included measures of heart rate variability in the time and frequency domains during supine rest (74), beat-to-beat finger blood pressure and heart rate responses to the Valsalva maneuver (31), and hemodynamic and plasma catechol responses to head-up tilt table testing for 5 minutes at 90 degrees from horizontal (32, 33).

### Efforts to reduce bias.

Determinations of eligibility for the study at the PDRisk webpage were done using a computer algorithm without regard to sex, race, or ethnicity. For the primary outcome measure, a neurologist who was blinded as to the reported biomarkers examined each participant at each follow-up visit and rendered a decision about whether the participant had PD, with a written consult note entered into the Clinical Research Information System. The personnel who analyzed the PET images, performed neurochemical assays, and conducted immunofluorescence confocal microscopy were blinded as to the identity of the participants until the data were tabulated in spreadsheets.

### Statistics.

Normality assumption testing and outlier identification were based on the studentized residuals. All *P* values are 2-tailed. The statistical analyses were performed using SAS version 9.4 (SAS Institute), Prism 9.3 for Mac OS (GraphPad), and KaleidaGraph 5.0 (Synergy Software). Statistical testing was done to answer the following questions.

(a) Does low ^^^18^^^F-dopamine–derived radioactivity increase the likelihood of a subsequent diagnosis of a central LBD? The survival time was defined as the follow-up years from the first visit date to the date when a central LBD was diagnosed, where all participants without LBD+ (including patients lost to follow-up) were considered censored. ^^^18^^^F-dopamine–derived radioactivity in the interventricular septum in the dynamic frame with midpoint about 8 minutes from initiation of tracer administration was dichotomized as low versus normal using a threshold of 6,000 nCi-kg/cc-mCi (24). Comparison of the time to LBD+ diagnosis between low and normal groups was performed using the Kaplan-Meier survival curve and log-rank test.

(b) Do LBD+ and LBD– groups differ in terms of initial ^^^18^^^F-dopamine–derived radioactivity? For each of the 6 biomarkers of ^^^18^^^F-dopamine PET at the time of initial evaluation, analysis of covariance (ANCOVA) with age and sex considered as covariates was applied to compare the means among participant groups (LBD+, LBD–, or HV), with Tukey’s method applied to the pairwise multiple comparisons.

(c) How well does low initial ^^^18^^^F-dopamine–derived radioactivity separate the LBD+ and LBD– groups? For each of the 6 initial ^^^18^^^F-dopamine PET variables, ROC curve analysis was used to assess the separability between the LBD+ and LBD– groups. The ROC curve AUCs were considered excellent (AUC ≥ 0.90), good (0.80–0.90), fair (0.70–0.80), or poor (<0.70). Sensitivity and specificity for each biomarker were estimated based on the optimal threshold chosen using Youden’s method.

(d) Does cardiac ^^^18^^^F-dopamine–derived radioactivity change during long-term follow-up in the LBD+ or LBD– groups? To examine changes over time in cardiac ^^^18^^^F-dopamine–derived radioactivity in the LBD+ and LBD– groups, a linear random coefficient model was used (76). The model was applied to evaluate the change in 8-minute radioactivity across follow-up years, where the intercept and slope were random effects and age and sex were considered as covariates. The linear model included time (follow-up years), group (LBD+, LBD–), and interaction of time × group. The interaction test was used to examine slope homogeneity between the groups.

(e) Do LBD+ and LBD– groups differ in terms of the uptake or subsequent loss of septal ^^^18^^^F-dopamine–derived radioactivity within the initial PET session? One way to separately examine denervation versus decreased vesicular storage is pharmacokinetic, by tracking the rapid early rise in ^^^18^^^F-dopamine–derived radioactivity and the relatively slow subsequent fall from the peak value across dynamic PET frames (48). Denervation would decrease the rate of accumulation of ^^^18^^^F-dopamine–derived radioactivity in the myocardium, because tissue uptake of the tracer depends on the plasma membrane norepinephrine transporter, which is missing in the setting of sympathetic denervation (77). Loss of cardiac sympathetic nerves would shift downward the curve relating ^^^18^^^F-dopamine–derived radioactivity to time across all the dynamic frames in a PET session, without affecting the rate of decline in radioactivity from the peak value. In contrast, decreased vesicular storage would accelerate the “washout” of radioactivity after the peak in ^^^18^^^F-dopamine–derived radioactivity (48).

A piecewise linear random coefficient model (76, 78) was used to examine the rapid increase and slow subsequent loss of ^^^18^^^F-dopamine–derived radioactivity in the LBD+, LBD–, and HV groups. The model with 1 breakpoint (representing 2 linear lines with different slopes) was applied to evaluate the difference in slope between the 3 groups (SAS procedure MIXED), where the intercept, the slope of the first linear line, and the difference in slope between the 2 linear lines were treated as random effects, and the covariance structure (3 × 3) was specified as unstructured.

(f) Are the 6 measures of ^^^18^^^F-dopamine–derived radioactivity and the 3 indices of central dopaminergic innervation correlated? Pearson’s and Spearman’s correlation coefficients were used to evaluate the relationship among the 6 biomarkers of dynamic ^^^18^^^F-dopamine PET and 3 biomarkers of central dopamine deficiency.

(g) Do the LBD+ and LBD– groups differ in terms of indices of central dopaminergic innervation or function upon initial evaluation? ANOVA (with multiple comparisons) among the LBD+, LBD–, and HV groups was conducted on data for CSF DOPAC levels (72), the PUT/OCC ratio of ^^^18^^^F-DOPA–derived radioactivity in the 15-minute static emission scan ending 2 hours after tracer administration (49), and the percentage decline in radioactivity between the first (beginning about 30 minutes after tracer injection) and second (ending about 120 minutes after tracer injection) 15-minute static emission scans (49).

(h) Does the LBD+ group differ from the LBD– groups in terms of physiological or neurochemical autonomic functions upon initial evaluation? Two-sample *t* tests were conducted comparing the LBD+ and LBD– groups with particular attention to heart rate variability in the time and frequency domains, baroreflex-sympathoneural and baroreflex-cardiovagal function, and hemodynamic and plasma norepinephrine responses to head-up tilt.

The main autonomic function variables for heart rate variability in the time domain were the interbeat interval (normalized by exclusion of ectopic beats or artifacts [NN]), the standard deviation of the NN interval, and the coefficient of variation of the NN interval. In the frequency domain the data were for low frequency (LF) power, high frequency (HF) power, normalized LF and HF power (LFnu, HFnu), and the LF/HF ratio (74).

Baroreflex-cardiovagal function was quantified by the slope of the relationship between cardiac interbeat interval and systolic blood pressure during phase II of the Valsalva maneuver (31) and by the magnitude of increase in heart rate for a given decrease in systolic pressure during head-up tilt (44). Baroreflex-sympathoneural function was quantified by the log of the baroreflex area in phase III after release of the Valsalva maneuver (79) and the log of the pressure recovery time (31).

A *P* value of less than 0.05 was considered significant.

### Study approval.

The IRB of the NINDS approved the PDRisk study in 2009. The study was conducted under NIH Clinical Protocol 09N0010, “Biomarkers of Risk of Parkinson Disease.” The setting was the NIH Clinical Center.

### Data availability.

Supporting data are in a supplemental Supporting Data Values Excel file.

## Author contributions

DSG designed the research study, conducted experiments, acquired and analyzed data, wrote and edited the manuscript, and generated figures and tables. CH contributed imaging data acquisition, tabulation, and analysis. PS contributed biochemical data acquisition, data analysis, and manuscript editing. GL performed neurological examinations, acquired and analyzed data, and edited the manuscript. JG provided patient care coordination, conducted experiments, and acquired and analyzed data. SM conducting experiments, acquired demographic and clinical data, and analyzed data. RI acquired and analyzed data and edited the manuscript. TW developed and applied statistical methods, analyzed data, generated figures and tables, and wrote and edited the manuscript. YS contributed to study conception and design and manuscript editing.

## Supplementary Material

Supplemental data

ICMJE disclosure forms

Supporting data values

## Figures and Tables

**Table 4:**
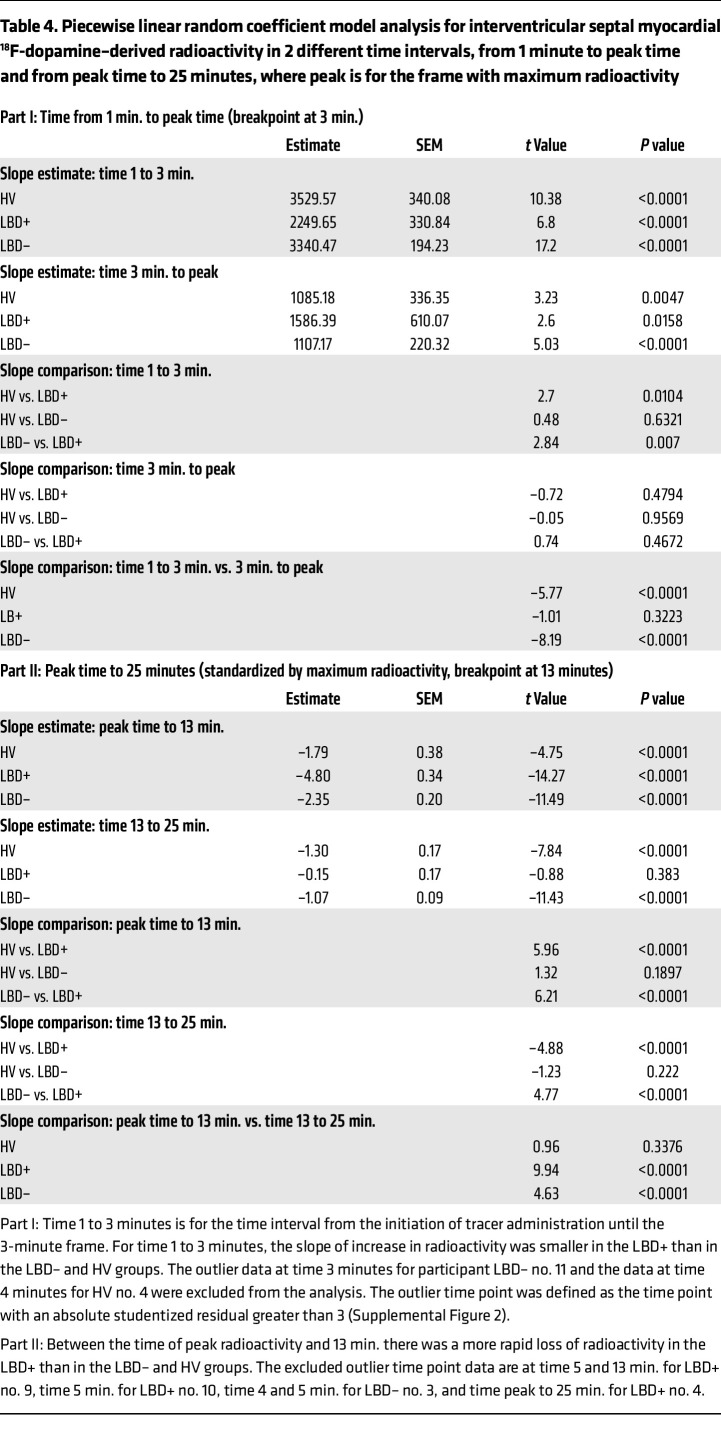
Piecewise linear random coefficient model analysis for interventricular septal myocardial ^18^F-dopamine–derived radioactivity in 2 different time intervals, from 1 minute to peak time and from peak time to 25 minutes, where peak is for the frame with maximum radioactivity

**Table 3:**
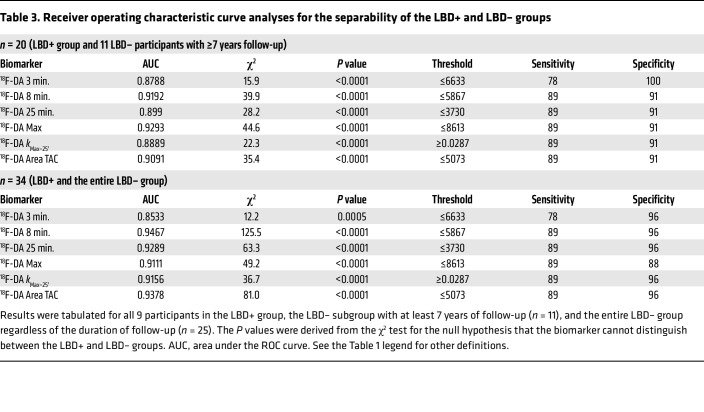
Receiver operating characteristic curve analyses for the separability of the LBD+ and LBD– groups

**Table 2:**
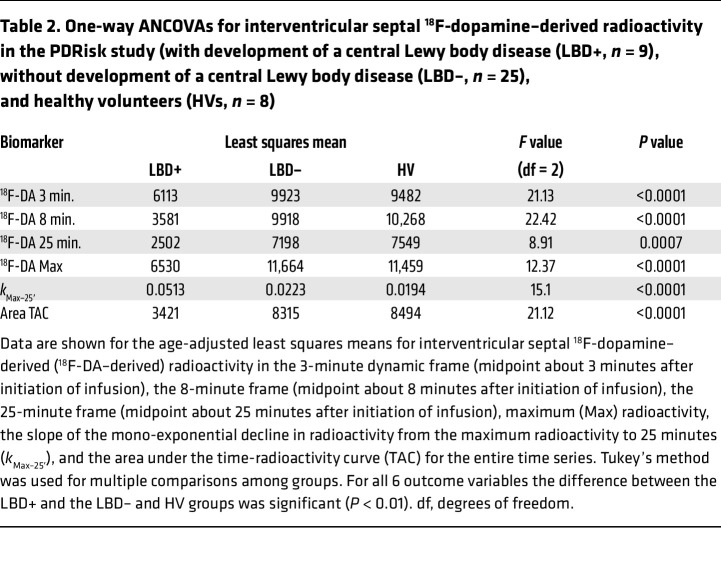
One-way ANCOVAs for interventricular septal ^18^F-dopamine–derived radioactivity in the PDRisk study (with development of a central Lewy body disease (LBD+, *n* = 9), without development of a central Lewy body disease (LBD–, *n* = 25), and healthy volunteers (HVs, *n* = 8)

**Table 1:**
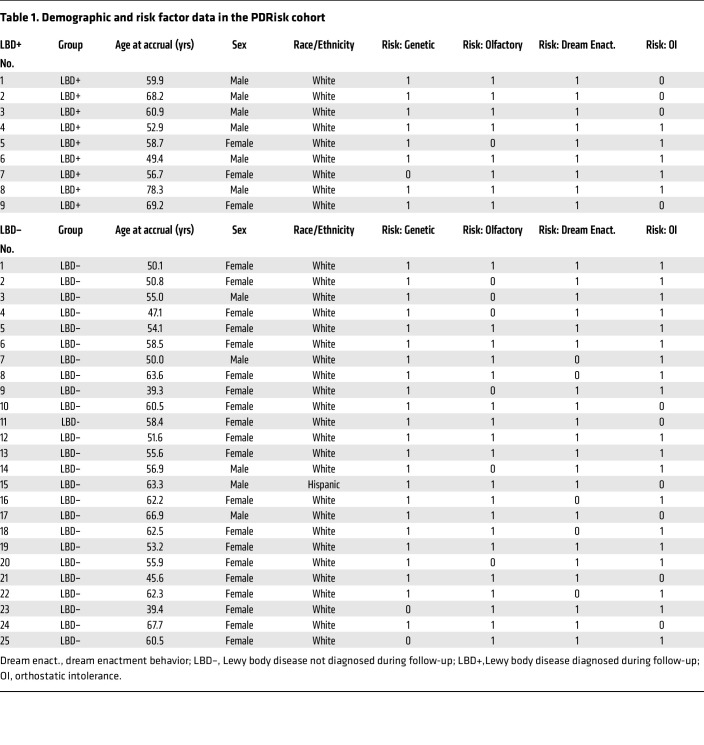
Demographic and risk factor data in the PDRisk cohort

## Version 1 10/26/2023

In-Press Preview

## Version 2 01/02/2024

Electronic publication
