# Supplementary material for: Cardiac noradrenergic deficiency revealed by 18F-dopamine positron emission tomography identifies preclinical central Lewy body diseases
Source: J Clin Invest. 2024 Jan 2;134(1):e172460. doi: 10.1172/JCI172460 (PMC10760969; doi:10.1172/JCI172460)
Supplement: Supplemental data [file jci-134-172460-s217.pdf]

Supplementary Table 1: Individual demographic, clinical, physiological, neurochemical, and neuroimaging data from the PDRisk study (PDRisk+, PDRisk-, HV; N=86).

Clinical Tab Abbreviations:

Betablk.=beta-adrenoceptor blocker; CNCS=Clinical Neurocardiology Section database; COMTI=catechol-O-methyltransferase inhibitor; CQ10=coenzyme Q10; DA Agonist=dopamine receptor agonist; FH/Occup.=family history/occupation; Florinef=fludrocortisone; HV=healthy volunteers; LBD-=Lewy body disease not diagnosed during follow-up; LBD+=Lewy body disease diagnosed during follow-up; MoCA=Montreal Cognitive Assessment; MRI=magnetic resonance imaging; MVI=multivitamin; Orth.=orthostatic; PDRisk-=less than 3 confirmed risk factors; PPI=proton pump inhibitor; Rx=medication; SSRI=selective serotonin reuptake inhibitor; UPDRS=Unified Parkinson Disease Rating Scale; UPSIT=University of Pennsylvania Smell Identification Test

Neurochemical Tabs Abbreviations:

Δ=change from baseline; BL=baseline; CSF=cerebrospinal fluid; DA=dopamine; DHPG=3,4-dihydroxyphenylglycol; DOPA=3,4-dihydroxyphenylalanine; DOPAC=3,4-dihydroxyphenylacetic acid; EPI=epinephrine; FxΔ=fractional from from baseline; LP=lumbar puncture; NE=norepinephrine; Ortho=orthostasis (head-up tilt).

Physiological Tab Abbreviations:

ΔBPd=change in diastolic blood pressure from supine rest to head-up tilt; ΔBPs=change in systolic blood pressure from supine rest to head-up tilt; ΔHR=change in heart rate from supine rest to head-up tilt; ΔMAP=change in mean arterial blood pressure from supine rest to head-up tilt; Baro. Area II=baroreflex area Phase II; Baro. Area III-IV=baroreflex area in Phases III-IV; Baro. Area Total=baroreflex area sum of Phase II+Phase III-IV; BPd=diastolic blood pressure; BPs=systolic blood pressure; CVNN=coefficient of variation of the cardiac interbeat interval; Fall in BPs Val=fall in systolic blood pressure from Phase I to the nadir pressure in Phase II of the Valsalva maneuver; HF=high frequency power; Inc. in HR Val=increase in heart rate from Phase I to the nadir blood pressure in Phase II of the Valsalva maneuver; LF Power=low

frequency power; LF/HF=LF power/HF power; MAP=mean arterial pressure; NN=cardiac interbeat interval; NU=normalized power; PRT=pressure recovery time; SDNN=standard deviation of the cardiac interbeat interval; SV Supine=cardiac stroke volume during supine rest; TP=total power; Val=Valsalva maneuver

Neuroimaging Tab Abbreviations:

03N0004=NIH Clinical Protocol 03N0004; 13NH3=^{13}N-ammonia; 18F-DA=^{18}F=dopamine; ANT=anterior; CAU:SN=caudate/substantia nigra ratio; CAU/PUT=head of the caudate/putamen ratio; CAUD/OCC=head of the caudate/occipital cortex ratio; CAUD=head of the caudate; CBL=cerebellum; Chamb.=left ventricular chamber; Eval.=evaluation; GP/PUT=globus pallidus/putamen ratio; GP=globus pallidus; k(Max to 25')=$k_{Max-25'}$; L=left; Max=maximum radioactivity; NH3=^{13}N-ammonia; OCC=occipital cortex; P/A=posterior/anterior ratio in the putamen; PET=positron emission tomography; POST=posterior; PUT:SN=putamen/substantia nigra ratio; PUT/OCC=putamen/occipital cortex ratio; PUT=putamen; R=right; SEPT or Sept.=interventricular septal myocardium; Static 1=15' static frame beginning 30' after initiation of ^{18}F-DOPA administration; SUB NIG/OCC=substantia nigra/occipital cortex ratio; SUB NIG=substantia nigra; Washout=percent decrease in putamen ^{18}F-DOPA-derived radioactivity between the first (30' from initiation of tracer administration) and second (15' frame ending 120' from initiation of tracer administration) static images.

Supplementary Table 2: Pearson and Spearman correlation coefficients for correlations among biomarkers of cardiac noradrenergic and central dopaminergic deficiency in the PDRisk cohort (LBD+, LBD-, HV; N=42).

Supplementary Figure 1: Kaplan-Meier plots for survival probability (i.e., lack of development of a central Lewy body disease) as a function of follow-up years in PDRisk study participants with low or normal initial cardiac ^{18}F-dopamine- (^{18}F-DA)-derived radioactivity.

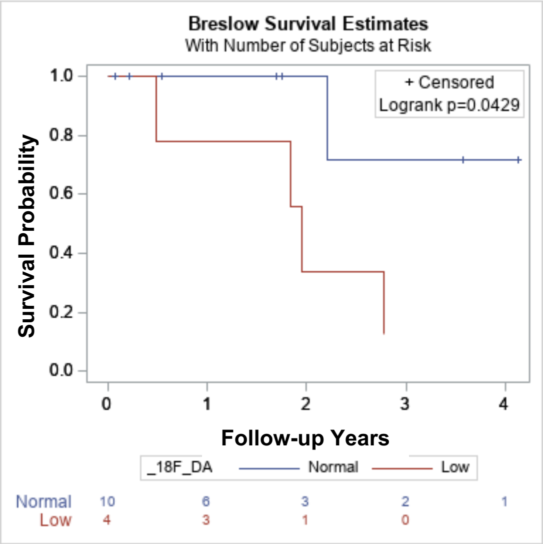

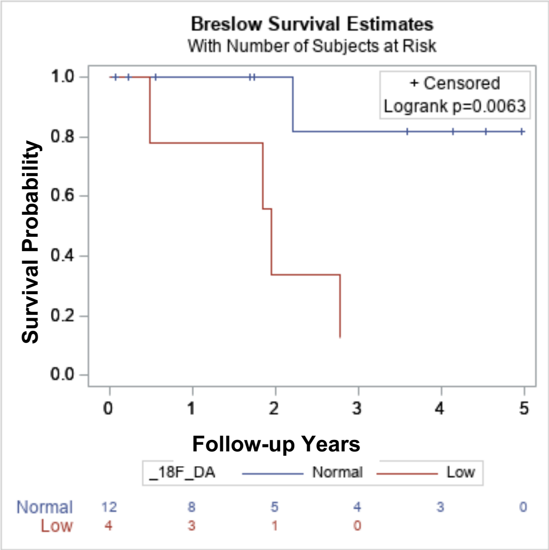

Left panel: log-rank test and Kaplan-Meier survival curve for subset with follow-up <4.5 years; Right panel: log-rank test and Kaplan-Meier survival curve for subset with follow-up <5 years

Supplementary Figure 2: Individual ^{18}F-dopamine-derived radioactivity vs. time curves for outliers that were not included in the breakpoint analyses.

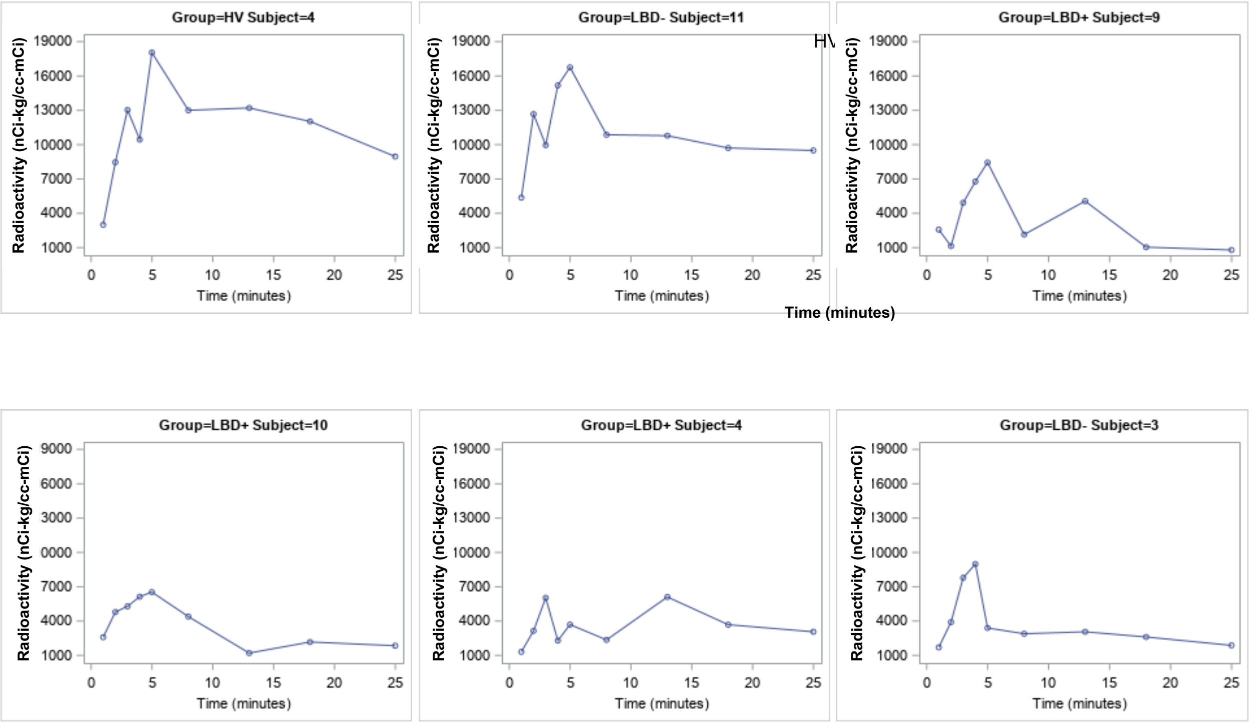

For the analysis in Part I, the data at Time 4’ for HV #4 and Time=3’ for LBD- #11 were excluded. For the analysis in Part II, the excluded outlier timepoint data are Time 5’ and 13’ for LBD+ #9, Time 5’ for LBD+ #10, Time 4’ and 5’ for LBD- #3, and Time peak to 25’ for LBD+ #4.

Supplementary Statistical Analysis Methods

The piecewise linear random coefficient model

Since ^{18}F-dopamine-derived radioactivity was not linear (increasing to peak and then decreasing from peak to midpoint 25'), the approximate low-rank smoother was applied to capture the trends over time using SAS procedure GLIMMIX. The Akaike information criterion (AIC) was used to determine the optimal number and location of breakpoints (interior knots) by changing the bucket size of the k-d tree. Three breakpoints at time=3, 5, 13 were constructed from the vertices of a kd-tree with the smallest AIC and best matched the expected with the observed radioactivity, in other words, a piecewise linear model with three breakpoints, which represents four linear lines with different slopes, fitted the radioactivity over the 25 minutes. However, the piecewise linear model with three breakpoints could not estimate the slopes for the second segment (time =3' to 5'). The main reason was that there were too few time points in the segment. Therefore, the radioactivity data were split into two parts: Part I included the data from 1' to the peak time (subject-based), and Part II included data from the peak time to 25'. Then, a piecewise linear random coefficient model with one breakpoint (at time=3' and 13') was applied to Part I and Part II data separately. In addition, some subjects with outliers were excluded based on the studentized residual analysis, their curve of the radioactivity within the initial PET also showed abnormal, for example, two subjects had two peaks.

In the piecewise (with one breakpoint) linear random coefficient model,

$$y = \beta_0 + \beta_1 t + \beta_2 \, \mathrm{I(t\text{-}k)} * \mathrm{t} + \beta_3 \, \mathrm{group} + \beta_4 \, \mathrm{group} * \mathrm{t} + \beta_5 \, \mathrm{group} * \mathrm{I(t\text{-}k)} * \mathrm{t} + \varepsilon$$

where y denotes the observed radioactivity

ε is the unobserved random errors

k is the breakpoint (k=3 for Part I data, k=13 for Part 2 data)

t is the time (t=1' to peak time for Part I, and t=peak time to 25 for part II data).

I(t-k) =0 for $t \leq k$; I(t-k) =1for $t>k$.

β_0 is the intercept and is treated as a random effect

β_1 is the slope for the first segment ($t \leq k$), and is treated as a random effect

β_2 is the difference in slope between the first ($t \leq k$) and second segment ($t \geq k$), and is treated as a random effect

β_3 is the group (with three levels: HV, LBD+, LBD-) effect and treated as fixed.

β_4 is the interaction between the group and β_1, which tests if the three groups have equal slopes in the first segment.

β_5 is the interaction between the group and β_2, which tests if three groups have equal difference in slope between the first and second segment. The test that the three groups have equal slopes in the second segment is constructed using the "estimate" option.
